# New Hepatocellular Carcinoma (HCC) Primary Cell Cultures as Models for Exploring Personalized Anti-TGF-β Therapies Based on Tumor Characteristics

**DOI:** 10.3390/ijms26062430

**Published:** 2025-03-08

**Authors:** Rosanna Scialpi, Rut Espinosa-Sotelo, Esther Bertran, Francesco Dituri, Gianluigi Giannelli, Isabel Fabregat

**Affiliations:** 1 TGF-β and Cancer Group—Oncobell Program, Bellvitge Biomedical Research Institute (IDIBELL), 08860 L’Hospitalet de Llobregat, Spain; rosanna.scialpi@irccsdebellis.it (R.S.); rutespinosa96@gmail.com (R.E.-S.); ebertran@idibell.cat (E.B.); 2 Medical Oncology Unit, National Institute of Gastroenterology, IRCCS “S. De Bellis” Research Hospital, 70013 Castellana Grotte, Italy; francesco.dituri@irccsdebellis.it; 3 National Biomedical Research Institute on Liver and Gastrointestinal Diseases (CIBEREHD), Instituto de Salud Carlos III, 28029 Madrid, Spain

**Keywords:** liver cancer, EMT, stemness, TGF-beta inhibitors, liver tumor spheroids, hepatic stellate cells

## Abstract

Transforming growth factor-beta (TGF-β) plays a dual role in hepatocellular carcinoma (HCC), acting as a tumor suppressor in early stages by inducing cell cycle arrest and apoptosis, and as a promoter in advanced stages by fostering tumor progression, epithelial–mesenchymal transition (EMT), and metastasis. Understanding TGF-β’s role in HCC progression, particularly its impact on tumor–stroma interactions, is crucial for developing personalized therapies. This study aims to clarify TGF-β function in HCC using patient-derived cell lines and advanced 2D and 3D culture models. Three new cell lines (HLC21, HLC19 tumoral, and HLC19 metastatic) were isolated from HCC patient biopsies, characterizing their phenotypic markers and responses to TGF-β and its inhibitor, galunisertib. HLC21 cells displayed a mixed epithelial–mesenchymal phenotype, responding to TGF-β suppressing growth and undergoing EMT, which were inhibited by galunisertib. Conversely, HLC19 tumoral and metastatic cells exhibited mesenchymal phenotypes and were resistant to both TGF-β suppression and galunisertib effects. In 3D co-cultures with hepatic fibroblasts, TGF-β inhibitory effects were diminished for responsive cell lines, while resistant lines maintained their non-responsiveness. These findings highlight TGF-β’s dual role in HCC and its influence on tumor–stroma crosstalk, offering valuable models for exploring personalized anti-TGF-β therapies based on tumor characteristics.

## 1. Introduction

Transforming growth factor-beta (TGF-β) is a pleiotropic cytokine that regulates a wide number of cellular processes, including cell proliferation, apoptosis/senescence, differentiation, and extracellular matrix production [1]. The TGF-β ligand binds to a type II receptor dimer, which recruits a type I receptor dimer, forming a complex with the ligand. These receptors are serine-threonine kinases. The type II receptor phosphorylates residues of the type I receptor, which activates its kinase activity. The main targets of this kinase activity are two members of the SMAD family: SMAD2 and SMAD3 which, once phosphorylated, recruit SMAD4 and are translocated to the nuclei. SMAD proteins regulate transcription with close ties to RNA polymerase II, in a highly orchestrated manner [2]. In the context of hepatocellular carcinoma (HCC), which is the most common form of liver cancer, TGF-β plays a dual role [3]. At early stages of hepatocellular carcinogenesis, TGF-β may inhibit tumor development by inducing cell cycle arrest and promoting apoptosis in hepatocytes [4,5]. However, as the tumor progresses, HCC cells often develop resistance to TGF-β growth-inhibitory and apoptotic effects through the activation of intracellular signals, such as the Epidermal Growth Receptor (EGFR) or the RAS/ERK pathways [6,7]. Once the tumor cells resist the TGF-β suppressor function, they may respond, undergoing epithelial–mesenchymal transition (EMT) and stemness [8], which may facilitate invasion of cancer cells into surrounding tissues and their spread to distant organs. TGF-β’s ability to alter the tumor microenvironment might also be critical for HCC progression. It induces the activation of hepatic stellate cells (HSCs) and fibroblasts, promoting fibrosis and the formation of a protumorigenic stroma [9,10]. Moreover, TGF-β modulates immune cell responses and recent evidence indicates that it may create an immunosuppressive microenvironment that further facilitates tumor growth [11,12].

Given TGF-β’s complex role in HCC, it was considered a target for therapeutic intervention [13]. Targeting the TGF-β signaling pathway by blocking its receptor kinase activity has shown promise in early-phase clinical trials [14,15]. However, the challenge remains to select those patients in which TGF-β may be contributing to cancer progression and effectively modulating TGF-β activity without disrupting essential normal physiological functions. Moreover, how TGF-β may influence the interaction between tumor cells and other non-tumoral cells in the microenvironment, such as fibroblasts, is not yet well understood. Although some works using commercial cell lines have been previously published, the isolation of cells directly from biopsies of HCC patients can improve the understanding of the TGF-β pathway situation in these patients at the moment of diagnosis and might help in the design of personalized therapies.

The aim of this work was to gain a more in-depth insight into the role of TGF-β in the progression of HCC by using new cell models obtained from HCC patients, cultured in 2D and 3D, alone or in combination with human hepatic stellate cells (fibroblasts) isolated from a healthy liver patient. The models are valuable for exploring personalized anti-TGF-β therapies based on tumor characteristics.

## 2. Results

### 2.1. Characterization of New Liver Tumor Cells Isolated from HCC Patients

**Figure 1:**
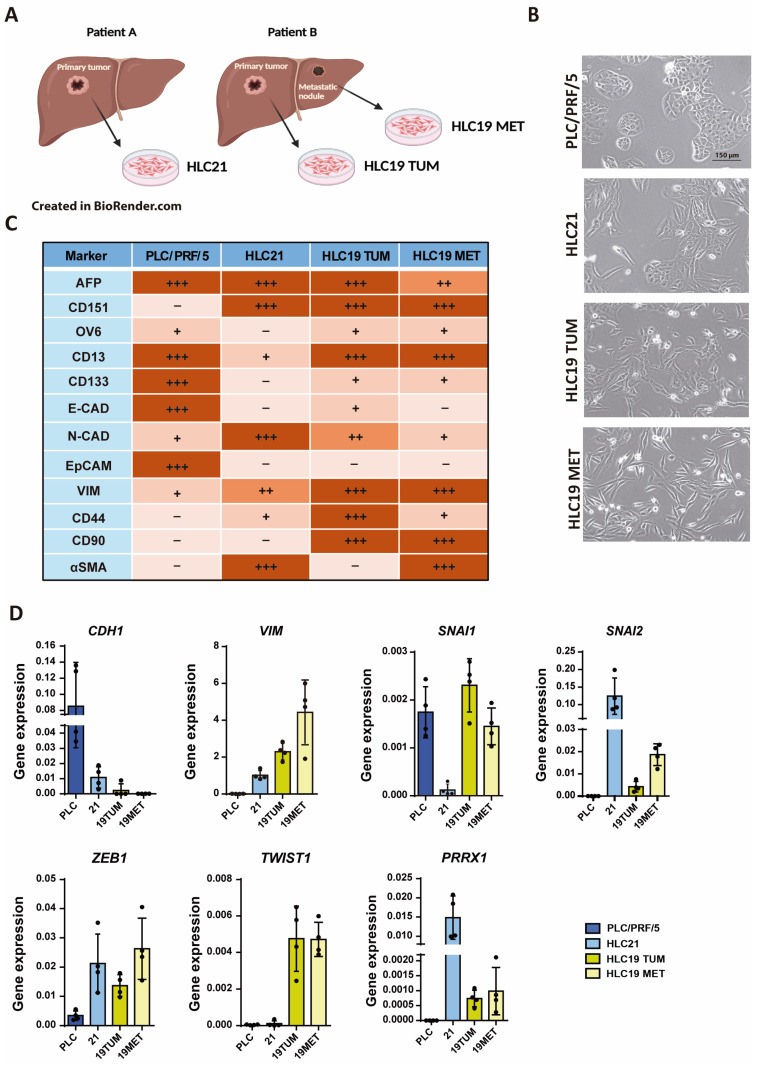
Characterization of new liver tumor cells isolated from HCC patients. (**A**) Scheme made by BioRender.com of the new cell lines isolated from HCC patient. (**B**) Phase-contrast images of PLC/PRF/5 and three new cell lines isolated from two patients. Bar: 150 µm. (**C**) Phenotypic analysis of epithelial–mesenchymal and stemness markers by flow cytometry, including PLC/PRF/5, that are well-known epithelial cells (− = 0% + < 30%; ++ = 30–70%; +++ > 70%). (**D**) Analysis of epithelial–mesenchymal and stem-related gene expression by RT-qPCR, standardized to the housekeeping gene *L32*.

We isolated liver tumor cells from two patients, denominated HLC21 and HLC19. In the case of HLC19, we obtained cells from the primary tumor and from an intrahepatic nodule (Figure 1A), from which were obtained HLC19 TUM and HLC19MET cells. The morphology of these cells in primary culture was compared with the morphology of the PLC/PRF/5 cell line, which has been widely used by our group and others as epithelial cells with a response to TGF-β in terms of growth inhibition (Figure 1B). The PLC/PRF/5 cells appear polygonal and more epithelial-like. They form clusters with some areas of dense cell–cell contact and the edges of the cells are defined. The HLC21 cells appear more elongated and spindle shaped. There is less clustering compared to PLC/PRF/5, indicating the transition to a potential mesenchymal or fibroblast-like phenotype. The HLC19 TUM cells are loosely arranged, and some cells display elongated extensions or protrusions, which may indicate motility; the overall structure suggests a less cohesive, migratory phenotype compared to tightly packed epithelial cells. The HLC19 Met cells appear clearly more elongated and scattered, with a strong mesenchymal morphology. There are fewer visible cell junctions, indicating a less cohesive structure, which may suggest a more migratory or metastatic potential.

A summary of their characteristics in terms of epithelial–mesenchymal and stem-related markers is shown in Figure 1C. We had previously characterized the HLC19 TUM [16] and here we characterized the HLC21 and the HLC19 MET cells, analyzing the presence and levels of the proteins by flow cytometry (Supplementary Figure S1A,B). The characteristics of the commercial PLC/PRF/5 cell lines were collected from previous publications [8,17,18,19]. The three new cell lines presented mesenchymal characteristics, when compared to the PLC/PRF/5, coincident with increased levels of vimentin (VIM) and CD151 (as a marker of EMT and invasive capacity [18], as well as expression of EMT-Transcription Factors (TF) (Figure 1D), such as *SNAIL2* or *ZEB1*. Interestingly, *TWIST1* expression was much higher in the HLC19 cells (both TUM and MET), whereas *PRRX1* was highly expressed in the HLC21 cells. The HLC19 cells presented the higher expression of mesenchymal stem proteins, such as CD44 or CD90. Alpha fetoprotein (AFP), characteristic of liver tumor cells, was expressed in all the cell lines. EpCAM, or CD133, epithelial stem markers, were mainly expressed in the PLC/PRF/5 cells. Expression of CD90, a stem-related protein more related with a mesenchymal phenotype, was high in the HL19 cells (both in TUM and MET) and expression of CD44 was mainly found in HLC19 TUM cells. Smooth Muscle Actin (SMA), a marker of activated fibroblasts or late EMT, was only expressed in the HLC21 and the HLC19 MET cells. Overall, the HLC21 cells presented the expression of mesenchymal proteins and EMT-TFs, such as *SNAIL2*, *ZEB1* or *PRRX1*, but low expression of stem markers. The morphology in culture reflected cell–cell adhesion capacity, forming parenchymal-like areas, which would be related to the high expression of N-cadherin (N-CAD) observed in these cells (Figure 1C). The HLC19 cells (both TUM and MET) also showed a mesenchymal phenotype, but coinciding with a high expression of mesenchymal stem proteins, such as CD13, CD90 or CD44 (in this last case, mainly in the HLC19 TUM).

### 2.2. Response to TGF-β in Terms of SMAD Phosphorylation, Growth Inhibition and Apoptosis

**Figure 2:**
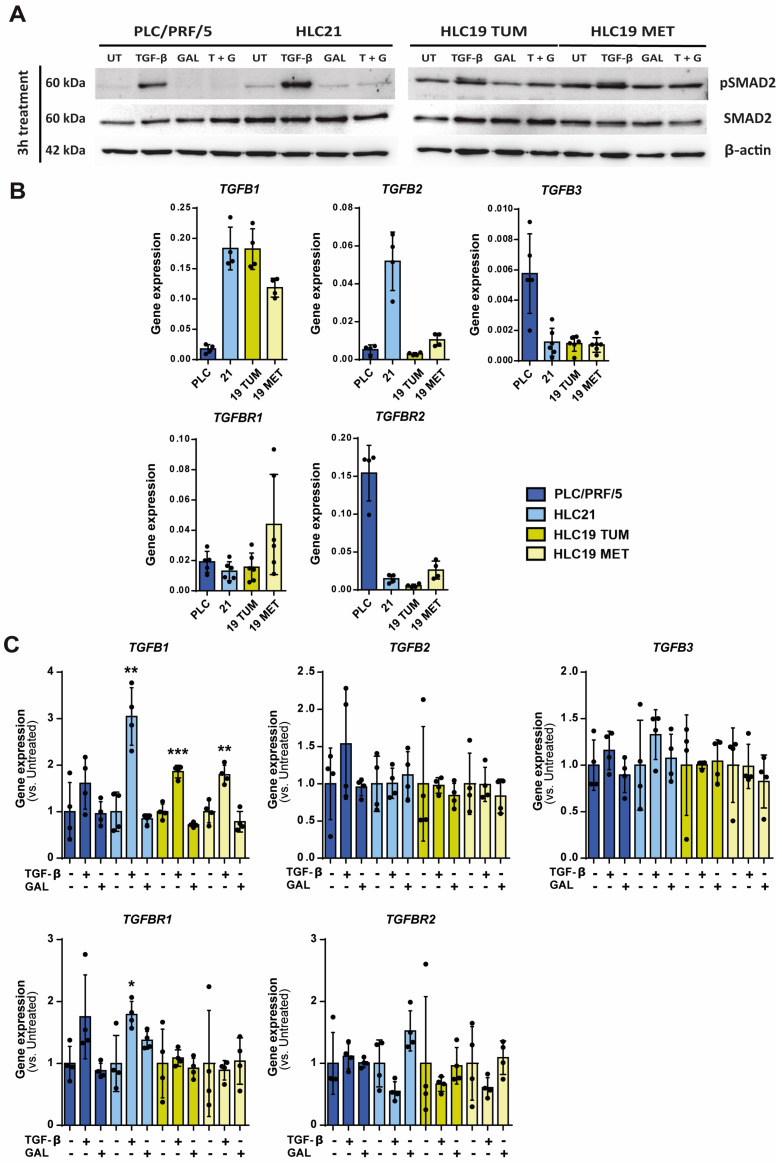
Response to TGF-β in terms of SMAD phosphorylation. (**A**) Cells were treated with TGF-β (2 ng/mL), TGF-β inhibitor galunisertib (10 µM) or both for 3 h. Analysis of SMAD2 phosphorylation by Western blot (a representative experiment, *n* = 3). (**B**,**C**) Cells were treated with TGF-β (2 ng/mL) or galunisertib (10 µM) during 48 h. Analysis of gene expression by RT-qPCR standardized to the housekeeping gene *L32*. In (**B**), basal levels. In (**C**), changes in gene expression following treatment with TGF-β and galunisertib, results expressed as fold of treated versus untreated: * *p* ≤ 0.05; ** *p* ≤ 0.005; *** *p* ≤ 0.001.

Phosphorylation of SMAD2/3 is the first response to TGF-β after the ligand-mediated activation of the kinase activity of the TGF-β receptor 1 [2]. Since the main purpose of this study was to analyze the response of these HCC cells to TGF-β or galunisertib (an inhibitor of the TGF-β Receptor1 activity), we started with an analysis of the cell response to TGF-β in terms of SMAD2 phosphorylation, to confirm that the selected concentration of galunisertib was adequate to inhibit its response. All the cell lines showed response to TGF-β in terms of SMAD2 phosphorylation (Figure 2A), which was inhibited by galunisertib. But it is noteworthy that all the HCC cell lines presented basal phosphorylation of SMAD2, which is more relevant in the HLC19 cells (both TUM and MET). A parallel experiment in which the Western blot was performed only in the cells untreated or treated with galunisertib and developed individually, to better visualize the effects, revealed that galunisertib inhibited the basal SMAD2 phosphorylation in all the cell lines (Supplementary Figure S2). This indicates that, together with the mesenchymal-like phenotype, these cells could be producing TGF-β in an autocrine way. Analysis of mRNA levels by RT-qPCR confirms high expression of *TGFB1* in the HLC21 and HCL19 cells (both TUM and MET), when compared to the epithelial PLC/PRF/5 cells (Figure 2B). HLC21 cells also presented a higher expression of *TGFB2*. Expression of *TGFB3* was very low in all the cells, although a little higher in the PLC/PRF/5 cells. The expression of the TGF-β Receptor 1 (*TGFBR1*) appeared to be higher in the HLC19 MET, but the expression of the TGF-β Receptor 2 (*TGFBR2*) was higher in the PLC/PRF/5 cells. In response to TGF-β, the most significant change was the increase in *TGFB1* and the decrease in *TGFBR2* mRNA levels in all the cell lines (Figure 2C).

**Figure 3:**
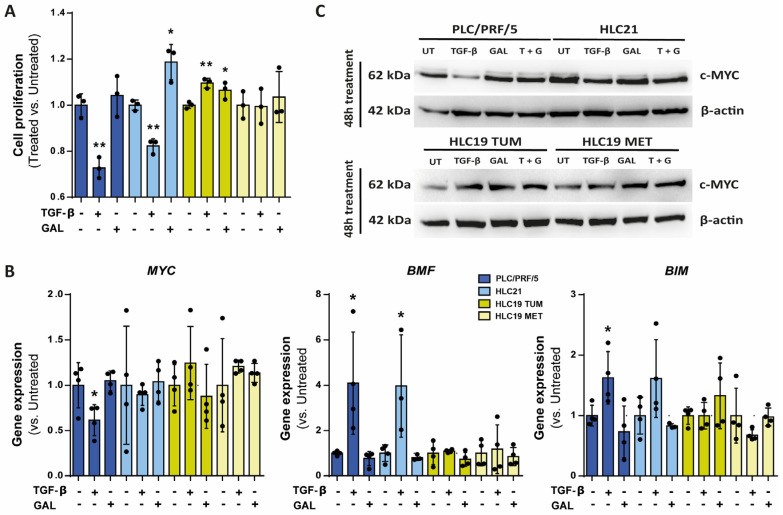
Response to TGF-β in terms of growth inhibition and apoptosis. Cells were untreated or treated with TGF-β (2 ng/mL) or galunisertib (10 µM) during 48 h. (**A**) Cell proliferation via analysis of viable cells by crystal violet staining. (**B**) Gene expression analysis of proliferation (*MYC*) and apoptosis (*BMF*, *BIM*)-related genes by RT-qPCR, standardized to the housekeeping gene *L32*. In (**A**,**B**), results are expressed as fold of treated versus untreated. * *p* ≤ 0.05; ** *p* ≤ 0.005. (**C**) c-MYC protein levels analyzed by Western blot (a representative experiment, *n* = 3).

Then, we focused our analysis to determine the response of these cells to TGF-β, whose concentration could be increased in the tumor, or to galunisertib, which could be a therapeutic option. The PLC/PRF/5 and the HLC21 cells responded to TGF-β by decreasing cell proliferation (Figure 3A), which was correlated with down-regulation of c-MYC both at the mRNA and protein levels (Figure 3B,C) and up-regulation of the pro-apoptotic genes *BMF* and *BIM* (Figure 3C). Treatment of cells with galunisertib had an impact on HLC21 cells, which proliferated more actively, and significantly decreased the expression of *BMF* and *BIM* (Figure 3B). This could indicate that inhibiting the autocrine activation of TGF-β in these cells might increase their proliferation and survival. To a lesser extent, galunisertib also had an impact in PLC/PRF/5 cells. However, HLC19 cells (both TUM and MET) did not decrease proliferation in response to TGF-β, which was correlated with a lack of response in terms of down-regulation of *MYC* expression (it even increased) or up-regulation of *BMF* or *BIM* (Figure 3A–C). Galunisertib had no impact in these cells.

### 2.3. Response to TGF-β in Terms of Cell Migration

**Figure 4:**
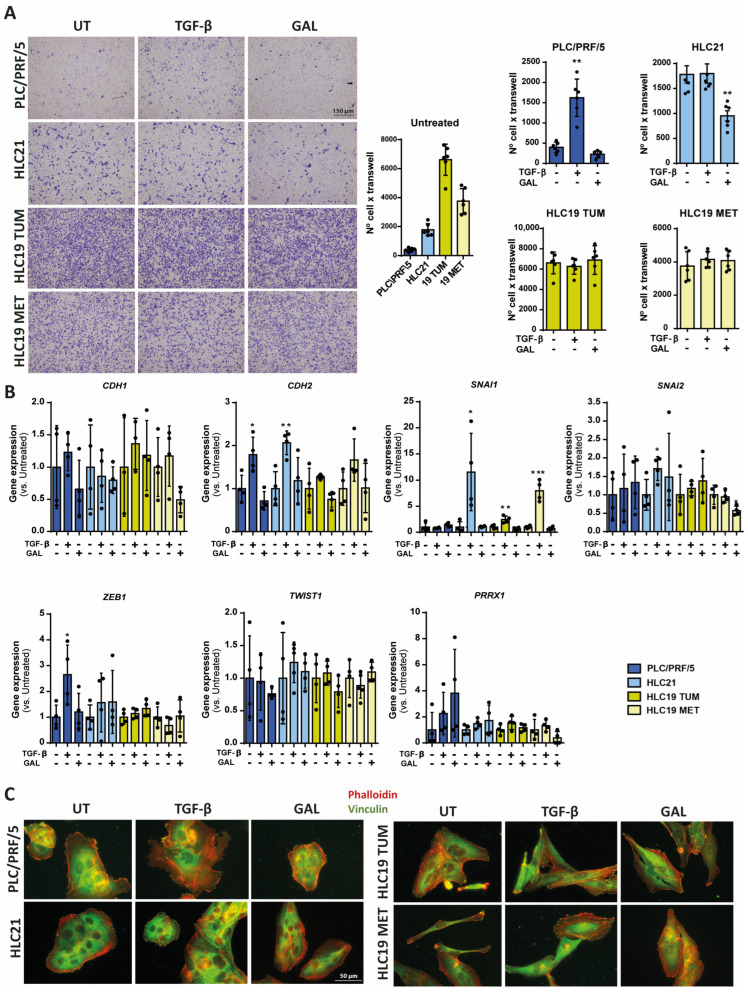
Response to TGF-β in terms of cell migration. Cells were untreated or treated with TGF-β (2 ng/mL) or galunisertib (10 µM) during 48 h. (**A**) Cell migration in two-chamber wells with a matrix of fibronectin between the two chambers. The cells that migrated were stained with crystal violet and the intensity analyzed in the images with Image J (version “v1.53k”). On the left, basal migration of untreated cells. On the right, response to TGF-β or galunisertib. (**B**) Gene expression analysis of epithelial–mesenchymal and stem-related genes by RT-qPCR, standardized to the housekeeping gene *L32* and expressed as fold of treated versus untreated: * *p* ≤ 0.05; ** *p* ≤ 0.005; *** *p* ≤ 0.001. (**C**) Immunofluorescence analysis of F-Actin (phalloidin) and focal adhesions (vinculin). A representative image is shown (*n* = 3).

When we analyzed the migratory capacity of the cells (Figure 4A), we observed that the HLC19 cells, particularly the HLC19 TUM cells, migrated more than the HLC21 and the PLC/PRF/5 cells. PLC/PRF/5 cells responded to TGF-β by increasing migration, but none of the three new HCC cell lines obtained from patients presented any increase in their migratory capacity in response to TGF-β. Interestingly, galunisertib prevented the migration of the HLC21 cells, but had no impact in the HLC19 cells (TUM or MET). E-cadherin (*CDH1*) expression did not show any significant difference following treatment either with TGF-β or galunisertib, but the expression of N-cadherin (*CDH2*), characteristic of mesenchymal cells, was increased by TGF-β in the PLC/PRF/5 and the HLC21 cells and decreased by galunisertib in the PLC/PRF/5 and the HLC19 TUM cells (Figure 4B). When analyzing the expression of EMT-TFs, we observed increased mRNA levels of *SNAI1* in response to TGF-β in all the cell lines, the greater increases being observed in the HLC21 and HLC19 MET cells (Figure 4B). The PLC/PRF/5 cells also showed increases in ZEB1 and the HLC21 cells increases in *SNAI2* mRNA levels after TGF-β treatment. None of the other EMT-TFs appeared to undergo major changes. In response to galunisertib, the most relevant result was the decrease in SNAI2 and PRRX1 observed in the HLC19 MET cells.

Finally, we analyzed the distribution of F-Actin in these cells, as well as the formation of focal adhesions, by immunostaining with phalloidin and vinculin. The response of PLC/PRF/5 cells to TGF-β was very clear, inducing changes in the cytoskeleton, stress fibers and focal adhesions (Figure 4C), an effect that was also observed in the HLC21 cells. The HLC19 TUM and MET cells presented a more mesenchymal-like structure for F-Actin, but TGF-β increased the appearance of stress fibers. The treatment with galunisertib appeared to increase the cell–cell contacts in the HLC19 (both TUM and MET), increasing the localization of F-Actin in the pericellular area.

### 2.4. Response to TGF-β in 3D Cultures

**Figure 5:**
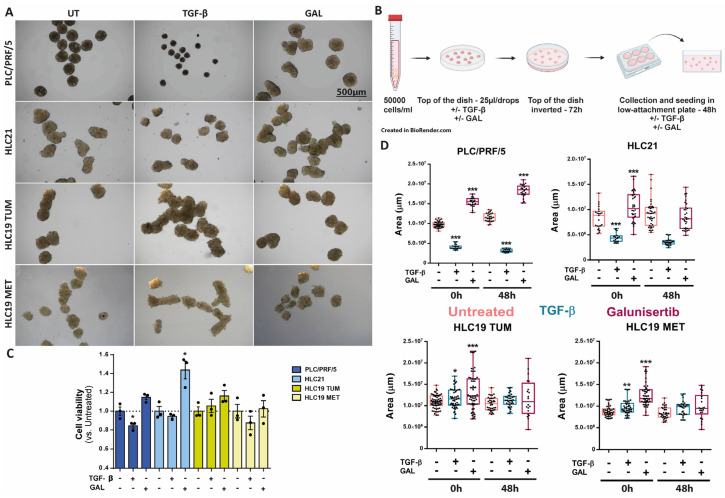
Response to TGF-β or galunisertib in 3D culture. Cells were untreated or treated with TGF-β (2 ng/mL) or galunisertib (10 µM) during the formation of the spheroids (72 h) and after seeding in low-attachment plates (48 h). Bar: 500 µm. (**A**) Representative photographs at the end of the treatment. (**B**) Scheme made by BioRender: formation process of the spheres and the treatment with TGF-β (2 ng/mL) or galunisertib (10 µM). (**C**) Analysis of cell viability in the spheroids, by MTS reagent. (**D**) Analysis of spheroid area using Image J. * *p* ≤ 0.05; ** *p* ≤ 0.005; *** *p* ≤ 0.001 treated versus untreated.

Since tumor cell response to extracellular cytokines is often conditioned by the microenvironment, we set up a protocol to culture these HCC cells in spheroids, which may allow the co-culture with other cells from the tumor environment. As presented in Figure 5A, all the cells can form spheroids. Treatment with TGF-β during the formation of the spheroids (72 h) and after seeding in low-attachment plates (48 h) (scheme in Figure 5B) produced a significant decrease in the cell viability and the size of the spheroids in the PLC/PRF/5 and the HLC21 cells, whereas in the case of the HLC19 (both TUM and MET) it had no significant effect on the cell viability and the spheroid size was even larger (Figure 5C,D).

The TGF-β receptor 1 inhibitor, galunisertib, induced a significant increase in the spheroid size in the case of PLC/PRF/5 cells both during the formation of the spheroid and over the next 48 h. In the HLC21 and HLC19 cells (both TUM and MET), galunisertib had an impact, increasing the size of the spheroids during their formation, but no differences were observed after the further 48 h of treatment. Neither TGF-β nor galunisertib had an impact on the circularity of the spheroids in any of the four cell lines analyzed (Supplementary Figure S3).

**Figure 6:**
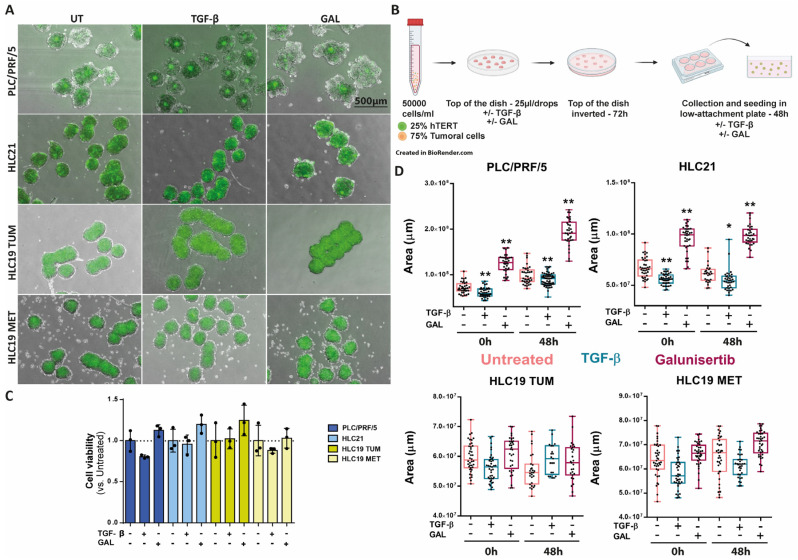
Response to TGF-β or galunisertib in 3D co-cultures of tumor and hepatic stellate cells. Cells (25% hTERT and 75% tumor cells) were untreated or treated with TGF-β (2 ng/mL) or galunisertib (10 µM) during the formation of the spheroids (72 h) and after seeding in low-attachment plates (48 h). (**A**) Representative photographs at the end of the treatment, in green the hTERT marked with a fluorescent cell tracker. Bar: 500 µm. (**B**) Scheme made by BioRender of the formation process of the spheres. (**C**) Analysis of cell viability by MTS reagent. (**D**) Analysis of spheroid area using Image J (version “v1.53k”). * *p* ≤ 0.05; ** *p* ≤ 0.005; treated versus untreated.

Next, we analyzed the formation and progression of the spheroids in the presence of hepatic stellate cells, the liver resident fibroblasts. For this, we used the immortalized cell line hTERT. The spheroids were formed with 25% hTERT and 75% tumoral cells in the absence or presence of TGF-β or galunisertib for 72 h. Once the spheroids were transferred to a low-attachment plate, they were untreated or treated for 48 h more (Figure 6A,B). In the presence of the hTERT cells, the response of the PLC/PRF/5 and HLC21 cells to TGF-β in terms of loss of viability was lower (Figure 6C, compared to Figure 5C), as well as the decrease in the size of the spheroids, but galunisertib continued to produce a very significant increase in the spheroid size (Figure 6D). In the HLC19 cells (both TUM and MET), no significant effect could be observed after either of the treatments.

## 3. Discussion

HCC remains one of the leading causes of cancer-related morbidity and mortality worldwide, posing significant challenges in both diagnosis and treatment [20]. Cancer immunotherapy has shown to be a promising method in treating cancer, but suboptimal responses in patients are attributed to cellular and molecular heterogeneity [21]. The inhibition of TGF-β activity has been proposed as a promising approach to increase the efficacy of T cell checkpoint blockade therapies [22]. However, recent advances in our understanding of HCC molecular pathogenesis have highlighted the complex and multifaceted role of TGF-β signaling in HCC development and progression [3]. This paradoxical behavior may underscore the use of TGF-β inhibitors in HCC. As the molecular landscape of HCC becomes increasingly well defined, it is evident that a one-size-fits-all treatment strategy is insufficient. Personalized therapy, tailored to the unique molecular and immunological characteristics of each patient’s tumor, is becoming a critical component in improving treatment outcomes. This paper has explored the TGF-β pathway, and the consequences of its inhibition, in HCC cells obtained from HCC patients, with the final aim of designing biomarkers that may facilitate personalized anti-TGF-β therapies based on tumor characteristics.

In this study, we incorporated HCC cells isolated from two different patients (HLC19 and HLC21). In one case, we obtained cells from the primary tumor area (HLC19 TUM) and from a later intrametastatic tumor (HLC19 MET). We compared their behavior with that of a well-known epithelial, non-invasive cell line, PLC/PRF/5. Overall, the results indicate that although all newly isolated cells from the patients exhibit some mesenchymal properties, their response to TGF-β and its inhibitor (galunisertib) differs significantly. HLC21 cells show a tumor-suppressive response to TGF-β, characterized by inhibited proliferation and induced apoptosis, that are correlated with down-regulation of cell-cycle-related genes and up-regulation of apoptosis-related genes. In line with this, galunisertib increases their proliferation in both 2D and 3D cultures. Results in terms of migration/invasion also revealed differences. The HLC19 cells (both TUM and MET) presented a higher migratory capacity than the HLC21 cells, and much higher than the PLC/PRF/5, as epithelial cells that do not migrate but induce cell migration in response to TGF-β. Any of these new HCC cells isolated from patients increased their migratory capacity in response to TGF-β, since they are already mesenchymal, but it was very interesting to observe that galunisertib was able to inhibit the basal migratory capacity of the HLC21 cells, correlated with a decreased expression of N-cadherin. However, even though an effect of galunisertib was observed in HLC19 (TUM and MET) in terms of increasing cell–cell adhesions, it did not exert any significant effect on their migratory capacity.

Examining the molecular and phenotypic characteristics of these cells, we observed clear differences in the expression of EMT-TFs. Notably, PRRX1 is highly expressed in HLC21 cells but much less expressed in HLC19 cells (both TUM and MET). High PRRX1 expression has been shown to significantly predict better overall survival in HCC patients, and PRRX1 knockdown accelerates proliferation and clonogenicity in HCC cell lines [23]. Furthermore, PRRX1 loss inhibits apoptosis in HCC cells and is accompanied by a down-regulated p53 expression. Indeed, HCC patients with low expression of both PRRX1 and p53 exhibit significantly shorter overall and disease-free survival [24]. Another key difference is the expression of TWIST1, which is highly expressed in HLC19 cells (TUM and MET) but absent in HLC21. TWIST1 regulates mesenchymal characteristics, such as vimentin expression, during the EMT of HCC cells [25]. Interestingly, HLC19 cells also show notable expression of stem-related genes, quite different from HLC21 cells, which exhibit a very low expression of stem markers, whether epithelial or mesenchymal. Low PRRX1 expression may contribute to the acquisition of cancer stem cell-like properties, as previously described [26].

Since tumor cell response is conditioned by the microenvironment, we also set up a protocol to culture these HCC cells in spheroids with or without other stroma cells, with a particular interest in fibroblasts, due to their relevant response to TGF-β [10]. It was interesting to observe that the drastic effect of TGF-β suppressing the growth of spheroids created with PLC/PRF/5 or HLC21 cells was attenuated in the presence of hepatic stellate cells, and correlated with a much lower effect on TGF-β-induced loss of cell viability. Previous works have demonstrated that a crosstalk may exist between hepatic stellate and HCC cells, which is regulated by TGF-β, through induction of different intracellular pathways, such as FAK signaling [27] that could inhibit HCC cell apoptosis. Hepatosphere formation by HCC cells proved to be enhanced upon co-culturing with cancer associated fibroblasts (CAFs) [28]. Altogether these findings indicate that to better understand the potential response of HCC cells to TGF-β in the patients, it is necessary to analyze the response in 3D cultures in the presence of other cell stroma components. Numerous studies have emphasized the significance of the interactions between HCC cells, CAFs, and other stromal cells in driving HCC progression. While both basic and clinical research have shed light on the emerging roles of CAFs in immunotherapy resistance and immune evasion, a deeper understanding of the specific functions of CAFs on HCC cells and tumor progression remains essential [29]. Nevertheless, despite the attenuation of the response to TGF-β in terms of inhibition of spheroid growth, galunisertib continued to increase spheroid growth in PLC/PRF/5 or HLC21 cells when the hepatic stellate cells were present. These results suggest some limitations in the use of galunisertib as a potential therapeutic option in these cases, although a potential role in inhibiting cell migration and metastasis could be expected. It should be noted that in the HCL19 cells (both TUM and MET), neither TGF-β nor galunisertib exerted any effect on the spheroid progression, not on HCC cells alone, nor in co-culture with hepatic stellate cells.

Altogether, these results indicate that some HCC patients may present tumor cells that are refractory to any response to TGF-β, as well as to its inhibitors, such as galunisertib. The EMT driven by TGF-β signaling promotes mesenchymal characteristics, including increased motility, invasion, and resistance to its pro-apoptotic activities [30]. However, our study indicates that HCC tumor cells exhibiting mesenchymal-like characteristics can show profound differences among patients, and that a low expression of PRRX1 and high expression of TWIST1, together with high levels of stem-related genes, is indicative of cells that do not respond to TGF-β-mediated tumor-suppressive responses, nor to inhibitors of the TGF-β pathway. Although we cannot exclude the possibility that TGF-β could have been responsible for the acquisition of a mesenchymal phenotype and stem-related characteristics in early phases of HCC in these patients [8] and that in some cases galunisertib could reverse these changes, as we previously described [31], our work reveals that in some patients these changes could be irreversible, probably due to epigenetic alterations [32] or microenvironment characteristics [33]. The relevance of this study was to establish some markers, in terms of gene/protein expression, which could anticipate the potential response of HCC patients to TGF-β inhibitory therapeutic approaches. Although the number of cell lines is limited, results reflect some of the differences that could exist in HCC patients. The molecular characteristics of the HLC19 TUM and MET cells were correlated with the lack of response to TGF-β or galunisertib. A similar molecular pattern found in an HCC patient would suggest that TGF-β inhibitors may be considered in combination with other therapeutic approaches, since they would not induce any effect on tumor cells but could be beneficial in inhibiting the protumorigenic function of TGF-β in the tumor microenvironment. Finally, our study clearly demonstrates the convenience of personalized therapy for the use of TGF-β inhibitors in HCC.

## 4. Materials and Methods

### 4.1. Cell Culture, Reagents, and Ethics Statement

PLC/PRF/5 cells were purchased from the European Collection of Cell Cultures. The cell lines HLC21, HLC19 tumoral and HLC19 metastatic were isolated from tumor and metastatic tissues, obtained from patients during surgical procedures at the IRCCS “S. De Bellis”, Castellana Grotte, Italy. Human cell lines were isolated and manipulated with the required informed consent in written from each patient and the approval of the Institutional Review Board. Patients’ written consent form and the study protocol conformed to the ethical guidelines of the 1975 Declaration of Helsinki. hTERT-HSC cells, derived from human hepatic stellate cells (HSC), were generated as previously described [34] and kindly provided by Dr. Lynda Aoudjehane (ICAN, Paris, France). All culture media and supplements were obtained from Gibco, Thermo Fisher Scientific (Waltham, MA, USA). PLC/PRF/5 HLC21, HLC19 tumoral and HLC19 metastatic cells were maintained in DMEM (Dulbecco’s Modified Eagle Medium), supplemented with 10% fetal bovine serum (Sera Laboratories International Ltd., West Sussex, UK), non-essential amino acids (Lonza, Basel, Switzerland), Amphotericin (2.5 μg/mL), Penicillin (100 U/mL), Streptomycin (100 μg/mL), and L-glutamine (2 mM). hTERT-HSC cells were cultured in DMEM 4.5 g/L glucose (31966-021) supplemented with the same reagent previously citated. All of them were maintained in a humidified atmosphere of 37 °C, 5% CO_2_. Cell lines were never used in the laboratory for longer than 4 months after receipt or resuscitation. TGF-β (#T70039, Sigma Aldrich, Saint Louis, MI, USA) was used at 2 ng/mL. Galunisertib (Eli Lilly and Company, Indianapolis, IN, USA) was used at 10 µM.

### 4.2. Isolation and Characterization of the Primary HCC Cell Lines

The assay was performed as previously described [16]. The primary HCC cell lines were collected from freshly tissue after surgical resection, HCC tumor sample were mechanically minced into 0.5–1 cm pieces and left in MACS Tissue Storage Solution (Miltenyi Biotec, Barcelona, Spain). The specimens were then finely chopped into smaller size pieces (1–2 mm), washed three times in Hanks balanced salt solution (HBSS), and then incubated in HBSS in the presence of type IV collagenase (Thermo Fisher Scientific) and 3 mM CaCl2 at 37 °C under gentle rotation for 4 h. After this step is completed, the dissociation was mechanically enhanced by pipetting the digested tissues up–down with a 50 mL pipette with a large-sized orifice. The floating cells were collected and washed three times with HBSS and seeded in normal culture conditions in DMEM + 20% FBS. The decanted, partially digested tumor sample were submitted to a second round of collagenase digestion. The isolated cells were washed with HBSS and cultured in DMEM + 20% FBS. The immunophenotypic characterization of cells was carried out after several (>10) culture passages, by using antibodies to detect epithelial markers (E-Cadh, EpCAM, AFP), mesenchymal markers (Vim, N-Cadh, αSMA), stemness markers (OV6, CD133, CD44, and CD90), and other cancer-related surface proteins (CD13, CD151).

### 4.3. Flow Cytometry

Cells were detached by using trypsin, then resuspended in PBS + 0.5% BSA + 0.1% sodium azide and incubated on ice in the presence of appropriate antibodies for membrane antigens staining. Alternatively, the detached cells were fixed and permeabilized using the Transcription Factor Staining Buffer Set (Ref. 00-5523-00, eBioscience—Thermo Fisher Scientific, Bremen, Germany), and then stained with the appropriate antibodies. After three washes the cells were finally resuspended in PBS + 0.1% sodium azide and analyzed using the Navios flow cytometer (Beckman Coulter, Miami, Florida, USA).

### 4.4. Cell Viability Analysis

#### 4.4.1. Crystal Violet Staining

The cells were cultured in 24-well plates. After treatment, the culture medium was removed; cells were washed with PBS and stained with crystal violet (0.2% (*w*/*v*) in 2% ethanol solution) for 35 min. The staining solution was washed with distilled water and dissolved with 10% SDS on a shaker for 35 min. Absorbance was detected on a 96-well plate reader at 595 nm.

#### 4.4.2. MTS Assay

Cell viability in spheroids was determined by an MTS assay with the kit CellTiter 96^®^ AQueus One solution Cell Proliferation Assay (#G3580A, Promega Biotech Ibérica S.L., Alcobendas, Madrid, Spain) This assay permits the evaluation of cell viability in a color detection method that can be detected in a spectrophotometer. At the desired time, 48 h, spheroids were collected and incubated in the working solution, as the manufacturer protocol established. For every 100 µL of media, 20 µL of reagent were added, and spheroids incubated for 2 h at 37 °C, 5% CO_2_. The absorbance was recorded at 490 nm in 96-well plates.

### 4.5. Transwell Migration Assay

Briefly, 2 × 10^4^ cells/well were seeded in 200 µL of the DMEM medium onto the top chamber of polycarbonate transwell inserts (suitable for a 24-well plate, a 6.5 mm internal diameter, and an 8 µm pore size, Corning, NY, USA), the membrane of which had been previously coated for 2 h at 37 °C with fibronectin (Fn at concentration 5 µg/mL) on the lower surface. Cells were allowed to migrate for 18 h in the DMEM medium at 37 °C and 5% CO_2_, then stained with crystal violet (0.2% (*w*/*v*) in 2% ethanol solution) for 35 min. The staining solution was washed several times with distilled water, and the microscopic images of the membrane were captured using the Leica Z16 APO vertical fluorescence stereomicroscope (Leica Microsystems, Barcelona, Spain), and the number of cells/transwell was counted.

### 4.6. Immunofluorescence and Image Acquisition

Cells were fixed with 4% paraformaldehyde and blocked using 1% BSA and 10% FBS in PBS during 1 h at RT. Primary antibody α-vinculin (Ref. V9131, Sigma Aldrich, Saint Louis, MI, USA) was diluted in 1% BSA in PBS (1:50 dilution, 1 h, RT), and Falloidin antibody (Phalloidin Red p1951 (vell) was diluted in 1% BSA in PBS (1:600 dilution, 1 h, RT). Samples were incubated with fluorescently conjugated secondary antibodies (anti-mouse Alexa 488, 1:200 dilution in 1% PBS-BSA, 1 h, RT). At the end, samples were embedded using ProLong™ Gold antifade reagent with DAPI reagent (Invitrogen #P36935, Thermo Fisher Scientific, Waltham, MA, USA). Cells were imagined in a Leica DM6000B vertical fluorescence microscope (Leica Microsystems, Barcelona, Spain).

### 4.7. Formation of 3D Spheroid Cultures

A particular solution was prepared for the formation of spheroid cultures. Four parts of the culture medium, supplemented by 2% of FBS, were mixed with one part of the medium with 1.2% of methylcellulose (Ref. M0512, Sigma Aldrich, Saint Louis, MI, USA), to obtain a final volume of 1 mL. Drops (25 μL) of cells were pipetted to the inside of a lid of a Petri dish (5 × 10^4^ cells for 1 mL in the final solution; 40 drops for dish; 1250 cells/drops for the PLC/PRF/5 and 1 × 10^5^ cells for 1 mL in the final solution; 40 drops for dish; 2500 cells/drops for the HLC21, HLC19 tumoral and HLC19 metastatic; +/−25% di hTERT cells), and carefully placed on the top of the dish filled with 5 mL cell culture medium. After 3 days incubation at 37 °C, 10% CO_2_, the spheroids (one spheroid formed per drop) were transferred by pipetting onto a 6-well low-attachment culture plate (Corning). A total of 40 spheroids from the 40 drops on one dish were placed per well. The spheroids were treated during the formation and after 48 h. For measuring the size (area μm) and circularity (arbitrary unit 1-0) of the spheroids, images were acquired with the Florescence microscope Olympus (Olympus España, Barcelona, Spain). The images of 10–20 spheroids per experiment were analyzed with software ImageJ (version “v1.53k”) using the same settings for all images.

### 4.8. Western Blot Analysis

Western blotting was carried out as previously described [10]. Cells were lysed in RIPA lysis buffer (supplemented with 5 μg/mL Leupeptin 1, mM PMSF, 0.1 mM Na3VO4, 20 mM β-Glycerol phosphate, 0.5 mM DTT) for 1 h at 4 °C. Protein was quantified with the Bio-Rad Protein Assay Dye Reagent Concentrate (Bio-Rad Laboratories, Hercules, CA, USA). Polyacrylamide gels were prepared at 10%. Primary antibodies were diluted in 0.5% non-fat-dried milk in PBS with 0.05% Tween 20 (c-MYC—ab32072 Abcam, Cambridge, UK; p-Smad2—S3108, Cell Signaling, Danvers, MA, USA; Smad2—S3103, Cell Signaling, Danvers, MA, USA). Peroxidase-conjugated secondary antibodies anti-mouse (NA931V) or anti-rabbit (NA934V) (GE Healthcare, Barcelona, Spain, 1/2000) were diluted in 0.5% non-fat-dried milk in PBS with 0.05% Tween 20.

### 4.9. Gene Expression Analysis

E.Z.N.A.^®^ Total RNA Kit II (Omega bio-tek, Norcross, GA, USA) was used for total RNA isolation following the manufacturer’s instructions. DMEM from the culture plates was removed, then the plates were washed with PBS and cells were scraped with RLT lysis buffer containing 20 μL/mL of β-mercaptoethanol. An amount of 1 μg of total RNA isolated from each sample was reverse-transcribed with random primers to synthesize complementary DNA, using a High Capacity RNA to cDNA Master Mix Kit (Applied Biosystems, Foster City, CA, USA), in accordance with the manufacturer’s guidelines. For the real time qPCR, expression levels were measured in duplicate in a LightCycler^®^ 480 Real-time PCR system, using the LightCycler^®^ 480 SYBR Green I Master (Roche Diagnostics GmbH, Mannheim, Germany). Gene expression derived from in vitro data was standardized to the housekeeping gene *L32*.

### 4.10. Statistical Analysis

The two-tailed *t*-Student test was used to measure the statistical significance for differences between the groups. *p*-values below 0.05 were deemed statistically significant. Overall, experiments were conducted at least three times independently. Data are represented as the mean ± standard deviation (SD). Statistical analyses were performed using GraphPad Prism software (version 6.01, 21 September 2012, GraphPad for Science Inc., San Diego, CA, USA). Differences were considered statistically significant at *p* < 0.05 (*), *p* < 0.01 (**) and *p* < 0.001 (***).

## Data Availability

The original contributions presented in this study are included in the article/Supplementary Materials. Further inquiries can be directed to the corresponding author(s).

## Supplementary Materials

The following supporting information can be downloaded at: https://www.mdpi.com/article/10.3390/ijms26062430/s1.
