# Supplementary material for: New Hepatocellular Carcinoma (HCC) Primary Cell Cultures as Models for Exploring Personalized Anti-TGF-β Therapies Based on Tumor Characteristics
Source: Int J Mol Sci. 2025 Mar 8;26(6):2430. doi: 10.3390/ijms26062430 (PMC11942228; doi:10.3390/ijms26062430)
Supplement: Supplementary file 1 [file ijms-26-02430-s001.zip › ijms-3479317-supplementary.pdf]

International Journal of
Molecular Sciences

MDPI

SUPPLEMENTARY FIGURES

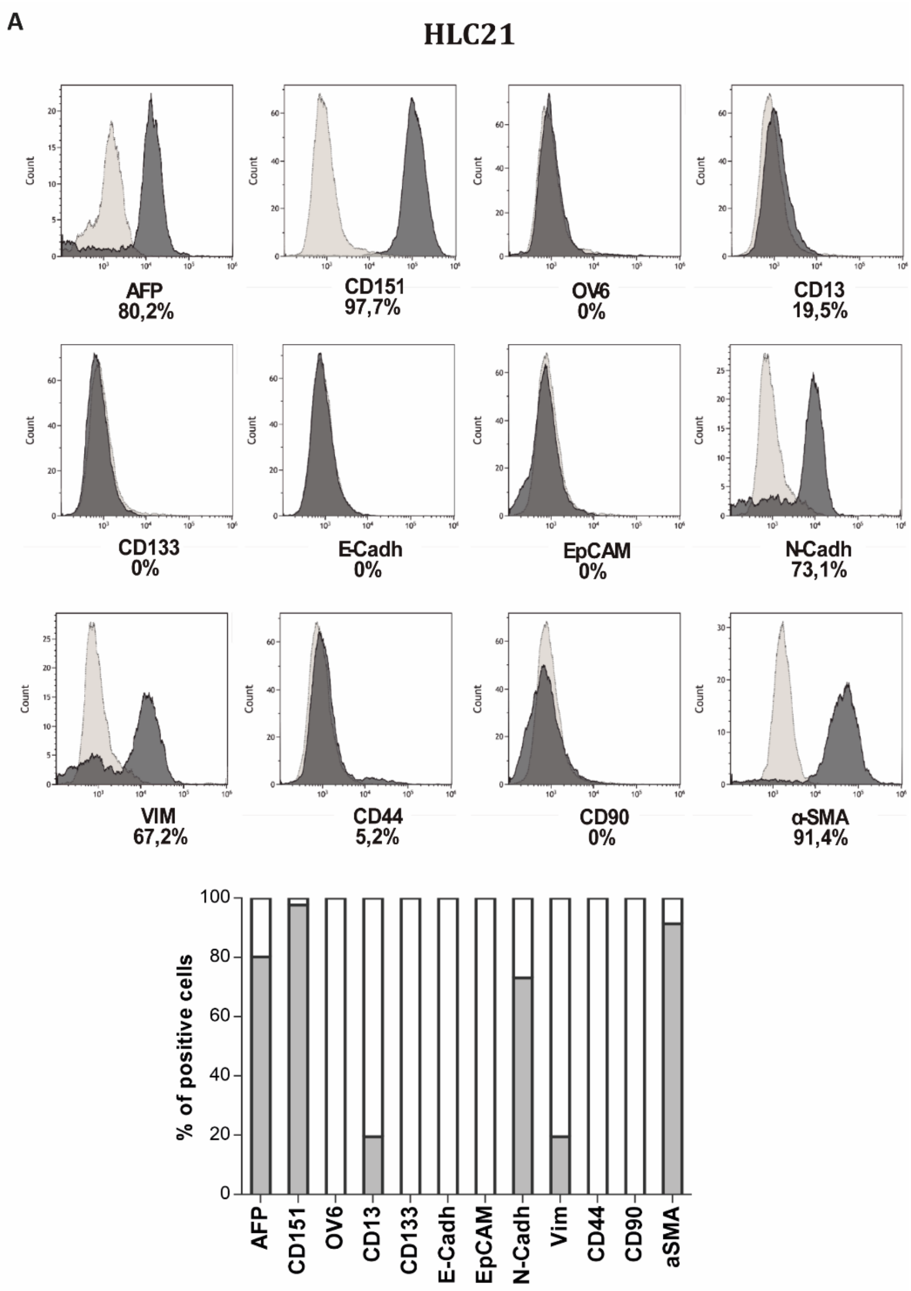

Supplementary Figure S1. Phenotypic analysis by flow cytometry of the new hepatic cancer cells isolated from HCC patients. Characterization of the new liver tumor cells HLC21 to analyze epithelial-mesenchymal and stemness-related proteins by flow cytometry. **A**: HLC21 cells.

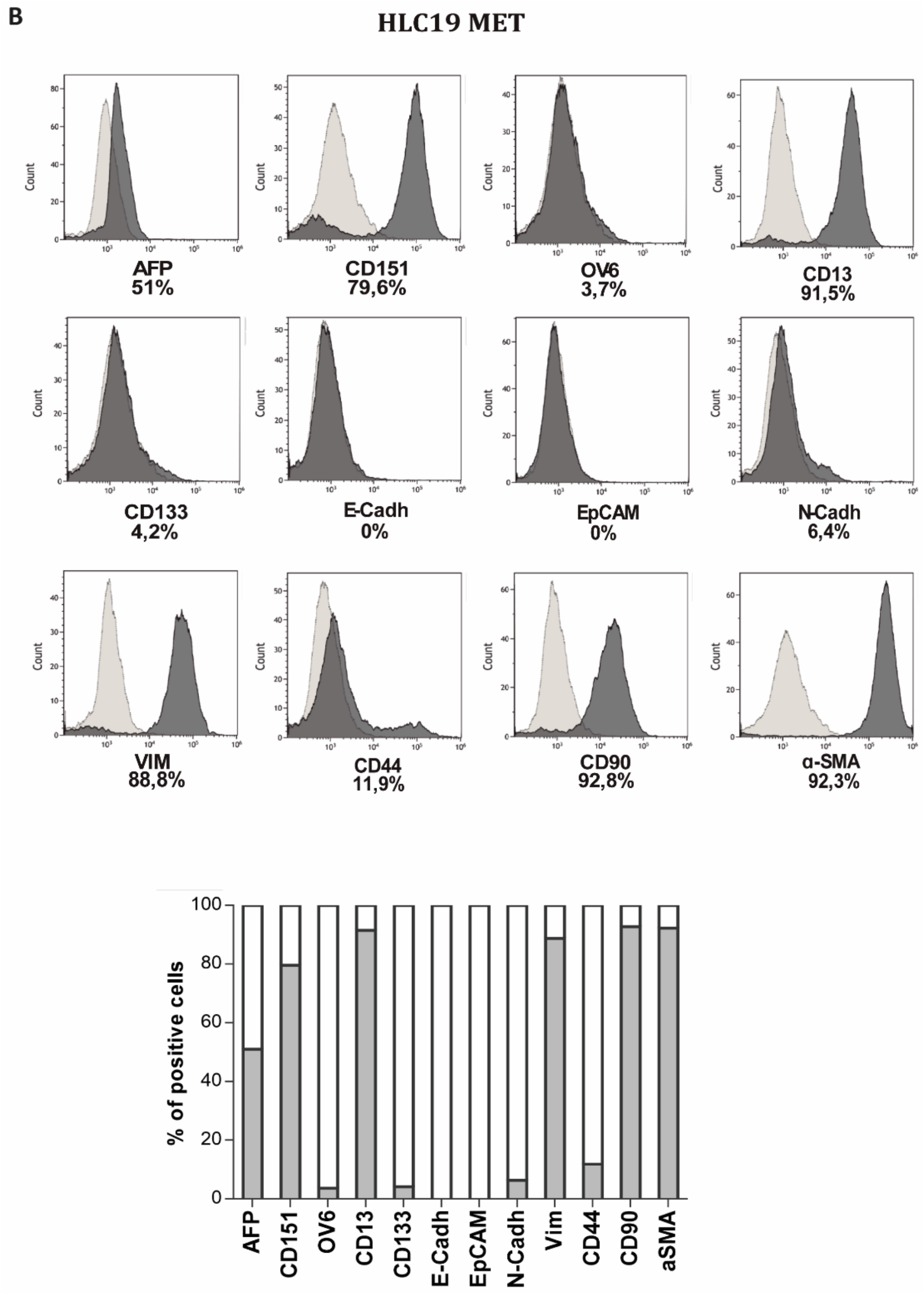

Supplementary Figure S1. Phenotypic analysis by flow cytometry of the new hepatic cancer cells isolated from HCC patients. Characterization of the new liver tumor cells HLC19 MET (metastatic) to analyze epithelial-mesenchymal and stemness-related proteins by flow cytometry. **B**: HLC19 MET cells.

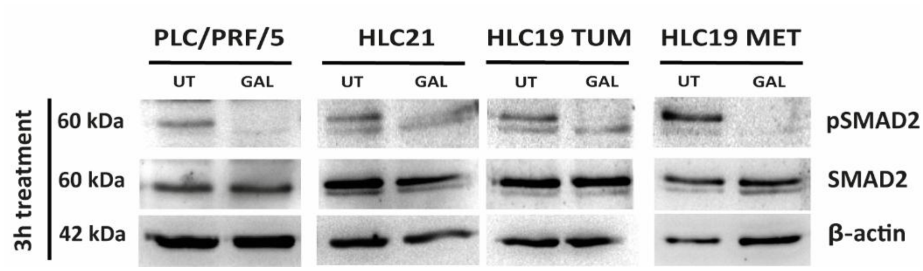

Supplementary Figure S2: Analysis of SMAD2 phosphorylation after galunisertib treatment. Cells were treated with TGF-β inhibitor galunisertib (10 μM) 3 h. Analysis of SMAD2 phosphorylation by western blot (a representative experiment, n=3).

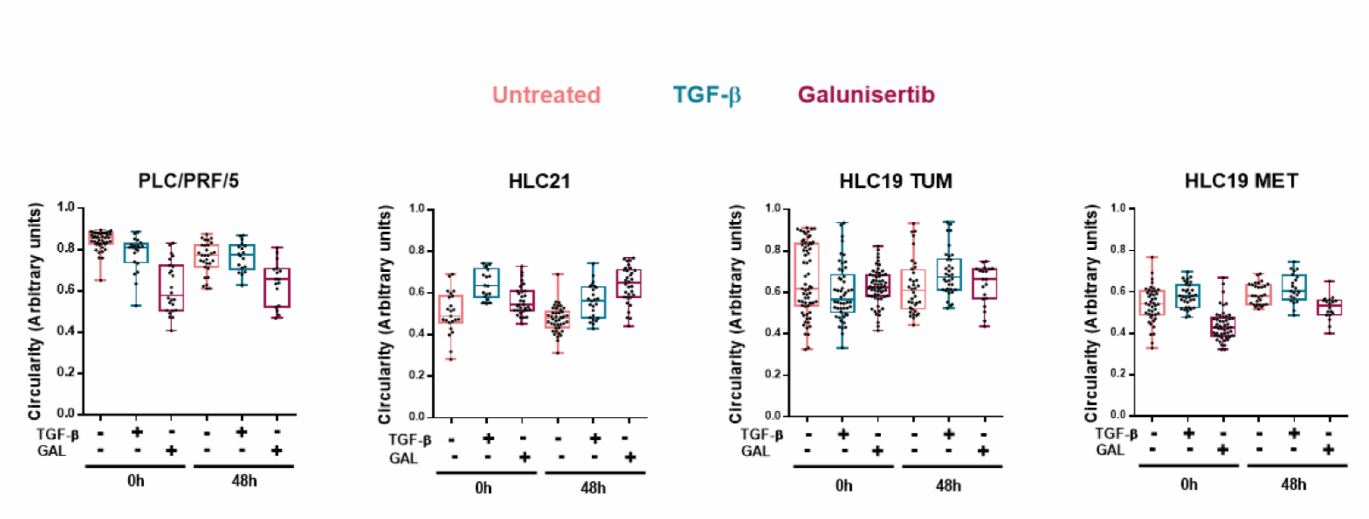

Supplementary Figure S3: Complement to the Figure 5: analysis of circularity. Cells were untreated or treated with TGF-β (2 ng/mL) or Galunisertib (10 μM) during the formation of the spheroids (72 h) and after seeding in low-attachment plates (48 h). Analysis of spheroid circularity using Image J.
